# Methodology for Extracting High-Molecular-Weight DNA from Field Collections of Macrofungi

**DOI:** 10.3390/jof11070490

**Published:** 2025-06-27

**Authors:** Leigh A. Burgoyne, Andy R. Nilsen, Teresa Lebel, Pamela S. Catcheside, Tom W. May, David Orlovich, Alan Kuo, Anna Lipzen, Kurt Labutti, Robert Riley, William Andreopoulos, Maxim Koriabine, Mi Yan, Vivian Ng, Igor V. Grigoriev, David E. A. Catcheside

**Affiliations:** 1 Biological Sciences, College of Science and Engineering, Flinders University, Adelaide 5001, Australia; leigh.burgoyne@flinders.edu.au (L.A.B.); teresa.lebel@sa.gov.au (T.L.); pam.catcheside@sa.gov.au (P.S.C.); 2 Department of Botany, University of Otago, Dunedin 9016, New Zealand; andy.nilsen@otago.ac.nz (A.R.N.); david.orlovich@otago.ac.nz (D.O.); 3 State Herbarium of South Australia, Adelaide 5000, Australia; 4 Royal Botanic Gardens Victoria, Melbourne 3004, Australia; tom.may@rbg.vic.gov.au; 5 U.S Department of Energy Joint Genome Institute, Lawrence Berkeley National Laboratory, Berkeley, CA 94720, USA; akuo@lbl.gov (A.K.); alipzen@lbl.gov (A.L.); klabutti@lbl.gov (K.L.); rwriley@lbl.gov (R.R.); william.andreopoulos@sjsu.edu (W.A.); mkoriabine@lbl.gov (M.K.); miyan@lbl.gov (M.Y.); vng@lbl.gov (V.N.); 6 Department of Plant and Microbial Biology, University of California, Berkeley, CA 94720, USA

**Keywords:** fungi, remote sampling, DNA extraction, chromatin, purification, long-read sequencing, genomes

## Abstract

Many macrofungi are impractical or impossible to culture. Consequently, DNA for long-read sequencing required for the assembly of high-quality genomes must be isolated from samples taken from the environment. Collection is often in remote locations, limiting the options for stabilising samples to methods that do not require refrigeration. Fungi contain species-specific arrays of metabolites that may complicate purification techniques and call for judgement to be made to apply appropriate modifications to the DNA extraction protocol in specific cases. The protocols and commentary we describe are informed by the preparation of DNA from a range of Australasian ectomycorrhizal and saprotrophic macrofungi. We collect samples into isopropanol at ambient temperature and employ a strategy of chromatin isolation followed by the sequential removal of unwanted molecular components to purify DNA.

## 1. Introduction

Ideally, fungal DNA is extracted from axenic cultures of mycelia. However, many fungi, particularly those involved in mycorrhizal associations, resist culture and DNA needs to be sourced from field-collected material. This typically involves extraction from differentiated tissue of sporing bodies (sporophores), which contain a species-specific range of metabolites, and exposure to the vagaries of environmental damage. Thus, fungal material collected from the wild is a highly variable starting material, presenting challenges for obtaining the high-quality DNA required for long-read sequencing. Due to this variability, no single procedure can be guaranteed to give clean, high molecular-weight DNA from fungi. The preparation of high-molecular-weight DNA from many organisms is well documented. Solvent methods mostly originate from Kay et al. [1], Kirby [2], and Marmur [3]. Methods based on binding to silica or glass derive from Boom et al. [4]. The preparation of small amounts of DNA from fungal tissue for PCR is a common task for which there are excellent commercial kits based on binding DNA to glass. However, once bound, high-molecular-weight DNA can be difficult to dislodge from surfaces, making it necessary to resort to the principles of solvent-based methods.

Besides its intrinsic chemical variability, a major issue with fungal material collected from the environment is the risk of low levels of apoptosis-like [5] or necrotic DNA damage following predation by insects, collembolans, worms, other fungi and bacteria. The resulting contamination with low-molecular-weight DNA, even when high-molecular-weight DNA remains, interferes with long-read sequencing. The methods described here allow yields of tens of micrograms of DNA supporting the application of pulse-field techniques to remove the offending short lengths of DNA. We present a protocol that generally enables good yields of high-molecular-weight DNA from the highly variable material of both mushroom-like and truffle-like fungi. The method is based on the isolation of chromatin, which can be washed free of many classes of cell metabolites, followed by the successive removal of residual carbohydrates, RNA, proteins, and other contaminants from the DNA, which is kept in solution. The protocol can be protracted but there are multiple points where the process can be interrupted to fit around other tasks.

## 2. Methods

### 2.1. Sample Collection

The absence of infrastructure in remote locations may make collection of tissue into liquid nitrogen or onto dry-ice impractical; therefore, other ways of stabilising samples for significant periods prior to DNA extraction are required. We adopted the approach of rapid dehydration of tissue slices by immersion in isopropanol to interrupt enzymatic degradation of DNA, along with paper strips impregnated with sodium carbonate to impede acid hydrolysis. The alternative method to protect DNA in the absence of freezing, based on the high-salt detergent CTAB [6], was not used because the way CTAB binds to DNA would disrupt chromatin [7].

Step 1.1: Up to ~4 g of tissue from young sporing bodies without obvious insect or other damage is sliced into a 50 mL Falcon tube containing ~40 mL isopropanol and a filter paper strip impregnated with sodium carbonate. Samples should only include internal tissue; where this is impractical, dirt and detritus are removed with a jet of high-quality water from a wash-bottle. There may be an advantage in avoiding hymenial tissue where this is practical, as recombination in meiotic cells could cause difficulties during genome assembly, in addition to those inherent in a dikaryon.

Step 1.2: Mix the collection tube contents by occasional inversion. In the field, random gyrations of collection bags suffice. Drain off and replace the isopropanol after 24 h. Keep the collections in a dark and cool environment, then at 4 °C on return to the laboratory.

Step 1.3: Drain off isopropanol and dry the sample under vacuum. Dried tissue can be stored at −80 °C.

It should be noted that the processing of tissues to purify DNA is best conducted in subdued light to minimise free-radical damage.

### 2.2. Tissue Disruption

**Figure 1:**
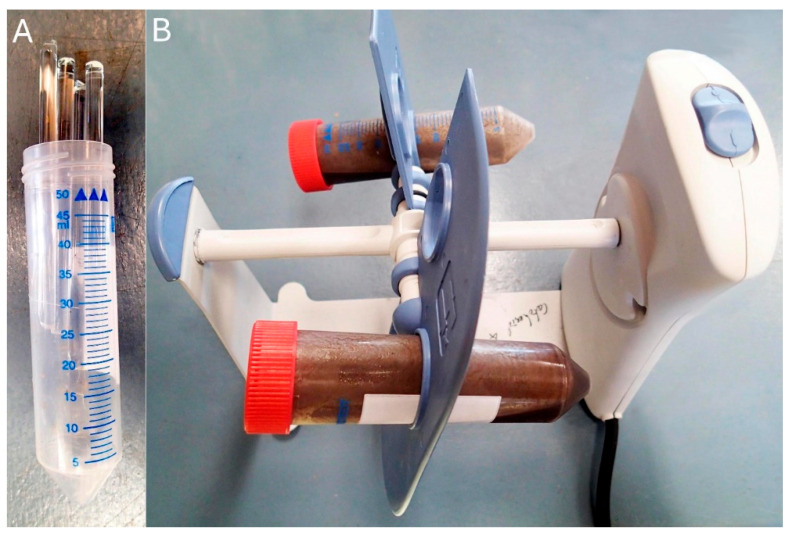
Tissue mill. (**A**) Falcon tube containing five 5 mm diameter glass rods, each slightly shorter than the tube, is loaded with 10 mL dry isopropanol and 200 mg dry fungal pieces, then closed with a lid and placed in a rotator, Labnet LNT H5600-50 (**B**), inclined at ~15° to the horizontal, rotating at 20 RPM for ≥48 h.

Step 2.1: Divide up to 200 mg of dry tissue into small pieces and mill (Figure 1) to a fine, smooth suspension in a falcon tube containing 10 mL isopropanol and five 5 mm diameter glass rods by slow rotation (~20 rpm) over two or more days at 4 °C. DNA in chromatin is not expected to be sheared significantly as it is still compact.

Step 2.2: Remove any residual lumps from the otherwise smooth suspension, centrifuge at 4000× *g* for 10 min at 4 °C, and discard the supernatant. Pellets can be vacuum-dried and stored at −80 °C if required.

### 2.3. Removal of Carbohydrate Polymers, Soluble Proteins, Soluble Polyphosphates, and RNA

Chromatin is stabilised by a buffer HAE containing polyamines, allowing it to be washed free of water-soluble tissue constituents.

Step 3.1: Resuspend pellet or 50–200 mg fungal powder (from step 2.2 above) in 10 mL HAE buffer by inversion and rotation at 20 rpm for 30–120 min at 4 °C. Break up any lumps using a wide bore plastic Pasteur pipette. Centrifuge ≥4000× *g* 10 min at 4 °C and discard the supernatant.

Step 3.2: Repeat wash step 3.1, breaking up any lumps. Washing can be extended until the supernatant is no longer turbid. We used three washes.

### 2.4. Digestion of Proteins and Residual RNA

Once released from chromatin, DNA is vulnerable to shearing and requires careful handling. Pipetting operations should be slow, using tips clipped to have an orifice ≥2 mm diameter. TTE inhibits DNAase activity. Digestions can be interrupted at any stage by freezing at −80 °C, preferably with tubes on their sides. To continue, thaw without shaking.

Step 4.1: Suspend pellets in 2.2 mL of a 1/10 dilution of TTE buffer in a 10 mL centrifuge tube by gentle pumping using a wide bore pipette.

Step 4.2: Add 60 µL heat-activated 3 mg/mL RNAase, mix by gentle inversion by hand and incubate at 37 °C for at least 1 h, or ideally 2 h.

Step 4.3: Add 10 µL fresh 10 mg/mL trypsin; mix by gentle inversion by hand and incubate undisturbed for a further 1 h, or ideally 2 h, at 37 °C.

### 2.5. Removal of Proteins and Cell Debris by Phenol Extraction

Following the trypsin treatment step, any mixing step should be carried out by gentle inversion to avoid shearing the DNA. We used a Ratek RSM7DC rotator at its lowest stable speed ~2 RPM. The digest from stage 4 is often a dark suspension. If stored frozen, warm to 37 °C before proceeding. Remaining proteins are denatured at the interface of a phenol emulsion and removed along with cell debris that form a pellet at the interface when the emulsion is broken by centrifugation. Check required safety precautions when using phenol to avoid skin or eye contact.

Step 5.1: Add 200 µL 5% SDS in 100 mM TTE and mix by gentle inversion by hand to yield approximately 0.4% alkaline SDS.

Step 5.2: Add 1 mL water-saturated phenol and mix for 1 h in a rotator (as specified above).

Step 5.3: Transfer the emulsion to microfuge tubes and centrifuge at 20,000× *g* for 30 min, at room temperature or preferably at 4 °C to reduce phenol concentration in the aqueous phase.

Step 5.4: Avoiding the interface, remove the upper aqueous phase into a 10 mL tube. If there is no obvious transparent phenol phase at the bottom of the tube, add more phenol and rotate again for a few minutes to remix then centrifuge again. The DNA yield can be improved by pipetting the boundary of the aqueous layer and interface into a new tube and centrifuging again to achieve an additional upper phase.

### 2.6. Isopropanol Precipitation and Triage

Step 6.1: Measure the volume of the aqueous phase from step 5.4 and layer on 1.5 volumes of isopropanol. Swirl gently to mix. If a fibrous semi-translucent precipitate forms, the preparation is likely to yield good-quality DNA (case A, proceed to 2.7). If the precipitate is fibrous and pasty white, there may be good-quality DNA accompanied by a large amount of carbohydrate (case B go to 2.8).

Step 6.2: If there is no visible precipitate, consider adding more isopropanol until a visible precipitate is formed and centrifuge at 1200× *g* for 15 min. The pellet is unlikely to contain high-molecular-weight DNA but may be suitable for PCR or further purification using a commercial silica column kit. If proceeding with this step, wash the pellet with ethanol, vacuum-dry it, and dissolve in 1 mM TTE or 10 mM TE buffer.

### 2.7. Case A

Step 7.1: If practical, allow the precipitate to cling to the tube wall, gently decant the supernatant, then invert the tube over absorbent paper to drain for 1 h. Otherwise, pellet the precipitate at low speed, 1200× *g* for 10 min at 4 °C and drain.

Step 7.2: Add 8 mL 70% isopropanol and leave to stand overnight at 4 °C to allow the phenol and fungal metabolites to diffuse out.

Step 7.3: Discard the supernatant, drain, add 5 mL dry isopropanol, and leave to stand overnight at 4 °C

Step 7.4: Discard the isopropanol and drain thoroughly. Add 1 mL 1 mM TTE and dissolve in the rotator at 4 °C in the dark. If it dissolves rapidly, the pellet is largely carbohydrate or low-molecular-weight DNA, in which case treat as in Case B (step 8 below). High-molecular-weight DNA takes longer to dissolve; 48 h or more.

Step 7.5: Centrifuge 1200× *g* for 10 min at 4 °C. Any pellet is probably carbohydrate and can be discarded. However, if copious, it may be worth processing as with Case B. Transfer the supernatant to a clean tube, add 1.5 volumes of isopropanol, drain the diaphanous precipitate and wash with at least one change of 70% isopropanol to remove traces of phenol.

Step 7.6: Redissolve in 600 µL 1 mM TTE by rotation as in step 7.4, allowing at least 48 h at 4 °C. Check after 24 h and break up any lumps using a wide bore Pasteur pipette. The higher the quality of the DNA, the longer it will take to dissolve. If the solution is turbid, centrifuge 1200× *g* for 10 min at 4 °C and discard any pellets. Proceed to Section 2.9. (polishing).

### 2.8. Case B

Step 8.1: Centrifuge 1200× *g* for 10 min. Drain the pellet and process as in steps 7.2 and 7.3.

Step 8.2: Discard the isopropanol and drain thoroughly. Add 0.6 mL 1 mM TTE and dissolve overnight as in step 7.4 above. Steps 8.3 and 8.4—to remove carbohydrate—should be completed in less than 2 h to minimise DNA damage by the acidic sodium acetate.

Step 8.3: Transfer to a microfuge tube and add 300 µL chloroform and 100 µL 3.0 M sodium acetate. Rotate as in step 5 above with end-to-end mixing or shake gently by hand to disperse the chloroform into an emulsion, then centrifuge 18,000× *g* for 30 min at 4 °C.

Step 8.4: Collect the supernatant into a clean tube, discarding the chloroform and any pellets. Layer on 2 volumes of dry ethanol and swirl gently to mix. If high-molecular-weight DNA is present, it will form a fibrous gelatinous precipitate. Collect on the tube wall or, if necessary, pellet by centrifugation 1200× *g* for 10 min at 4 °C. Wash with 70% ethanol in water and vacuum-dry to remove any traces of acetic acid until odourless to ensure any residual acidity is controlled. Dissolve in 600 µL 10 mM TTE as in step 7.4.

Step 8.5: Add 900 µL isopropanol, mix gently by hand and centrifuge at 1200× *g* for 10 min. Discard the supernatant, vacuum-dry, and redissolve in 600 µL TTE as in step 7.4, then proceed to Section 2.9. (polishing).

### 2.9. Polishing

Any remaining carbohydrate contamination can usually be removed by repeating isopropanol precipitation using the minimum amount of isopropanol possible and by drying the precipitate. This takes advantage of any differential solubility of the carbohydrate from that of DNA and the tendency of carbohydrates to cross-esterify or otherwise cross-link under dry conditions, allowing the DNA to escape the insoluble mass by charge repulsion [8,9]. If the solution is coloured and the colour coprecipitates with DNA then dialysis can be used (Section 2.10).

Step 9.1: Add 750 µL isopropanol, rotate by hand to mix and leave at 4 °C in the dark for an hour. If all has gone well, a curd will appear. Collect it by centrifugation at 20,000× *g* for 1 min or by “spooling” onto the tip of a Pasteur pipette.

Step 9.2: Dry the pellet under vacuum, redissolve in 0.5 to 1.5 mL of 1 mM TTE, and check the spectrum by nanodrop. If the solution has colour, consider further polishing by dialysis.

### 2.10. Removal of Coloured Compounds

Coloured ionic compounds that co-precipitate or bind to DNA can commonly be removed by dialysis at high ionic strength.

Step 10.1: Boil dialysis tubing in deionised water and wash in multiple changes. Knot the tubing once, add the DNA solution, and knot again to close. Suspend the bag in 50 mL 0.1 M sodium phosphate buffer pH 7.6, 1 mm EDTA containing 1 g activated charcoal (Sigma Aldrich, St. Louis, MO, USA, cat no 31616) and a magnetic stir bar. Dialyse either overnight or longer at 4 °C with sufficient stirring to suspend the charcoal.

Step 10.2: Recover the dialysis bag, rinse and suspend it in 50 mL 1 mM TTE or another preferred buffer overnight at 4 °C.

Step 10.3: Recover the DNA and check the spectrum by nanodrop.

### 2.11. Storage and Shipping

DNA was stored in the dark at −80 °C. For shipping at ambient temperature by air courier, DNA was mixed with DNAstable^®^ Plus and processed according to the manufacturers’ instructions.

## 3. Results

**Figure 2:**
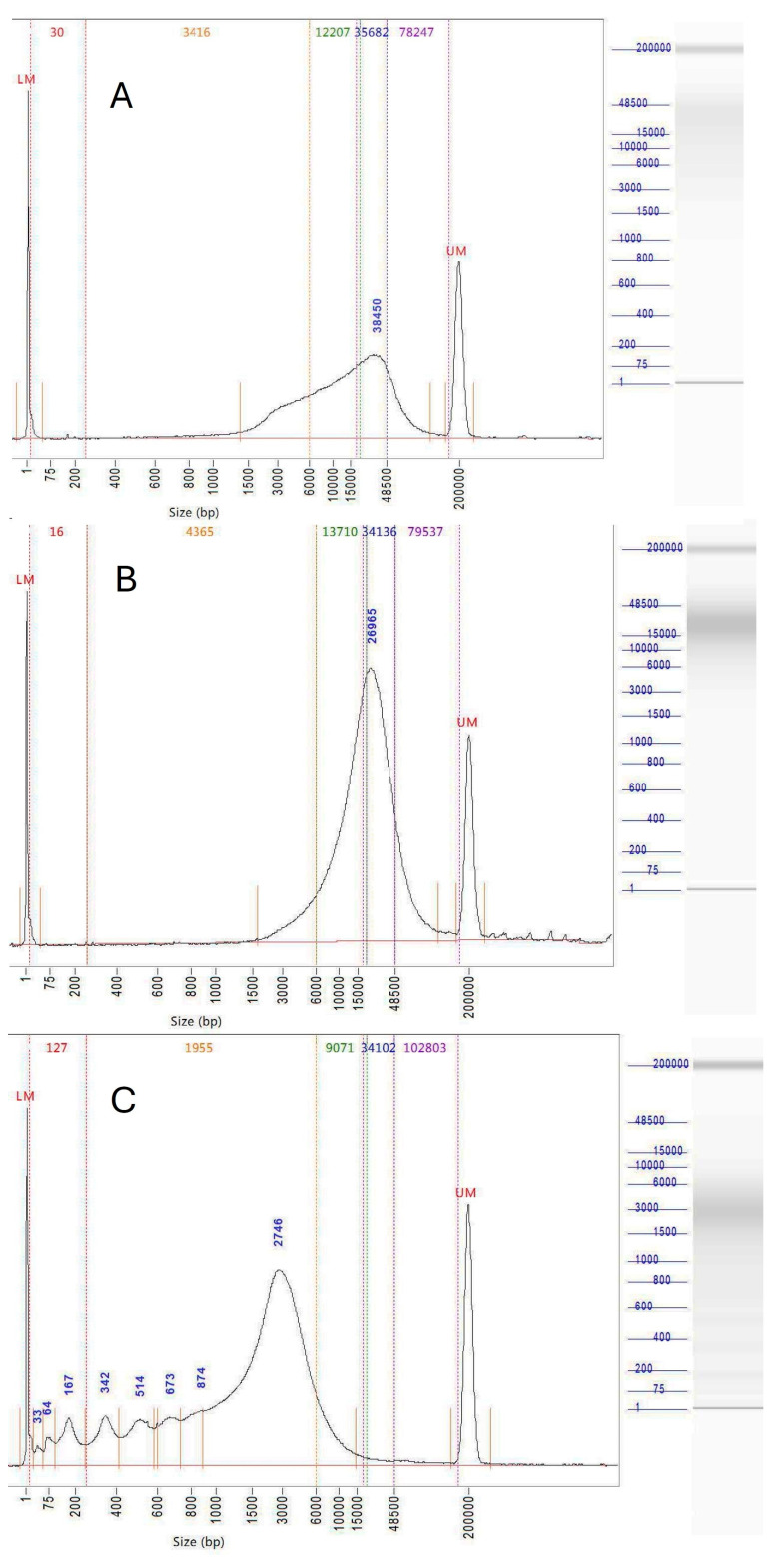
Capillary electropherograms of exemplar DNA preparations made by the methods described here. (**A**) *Cortinarius austrovenetus* BRI184, peak 38,450 bp, (**B**) *Laccaria* sp. LAT2 KIS6, peak at 26,965 bp and (**C**) *Cortinarius* [*Thaxterogaster*] aff. *campbelliae* 2 KIS2, showing ~200 bp ladder of peaks typical of enzymatic digestion of chromatin during apoptosis-like processes and necrosis.

A total of 64 preparations of DNA were made from 48 collections from nature of 33 species of macrofungi (Table 1) and from seven species cultured by us, yielding DNA suitable for long-read sequencing of 23 different genomes. Modal molecular lengths up to 38,000 bp were obtained in DNA preparations from field-collected fungi (Figure 2). Genome quality measured by either CEGMA or BUSCO odb10 scores ranged from 97.8% to 99.6% and 95.5% to 98.9%, respectively. Cultured mycelium from saprotrophic species (*Clavogaster virescens, Clavogaster* sp. ‘whakapapa’, *Leratiomyces ceres, L. erythrocephalus*, *Psilocybe subaeruginosa* and *Stropharia rugosoannulata*) as well as the single culturable ectomycorrhizal fungus *Cortinarius* [*Thaxterogaster*] aff. *campbelliae,* each yielded DNA preparations that passed JGI quality control. From those species that could not be cultured, ten DNA preparations passed quality control and seven required pulse-field electrophoretic removal of contaminating low-molecular-weight DNA to be useable. For samples containing good-quality DNA, 200 mg of dry fungus gave yields ranging from 25 to 80 µg of DNA. Exceeding 200 mg of dry fungus was counterproductive and extraction efficiency was improved when less dried material was used. *Laccaria* sp. LAT2 cap tissue yielded nearly five times more DNA per milligram of dry fungus than stem tissue.

Repeat sampling of material from the same collection yielded DNA suitable for long-read sequencing in five of 11 trials, though three of these required pulse-field electrophoresis to reduce contamination by short molecules. Where the collection of new material was possible, useable DNA was obtained from six of the ten species. However, five collections were needed in the extreme case of truffle-like *Laccaria* [*Hydnangium*] aff. *carneum* to obtain a single preparation suitable for long-read sequencing. The success of the methodology was dictated by the source of the DNA; all seven preparations made from species that could be cultured yielded genomes, while samples from collections from nature were of sufficient quality for the assembly of a high-quality genome in only 16 of 41 preparations (Fisher exact *p* = 0.0004). There was no significant difference (Fisher exact) in the rate of success or failure between different families (*p* = 0.3301) or morphologies (*p* = 1.0) of these fungi. Electrophoresis of failed preparations showed a varied extent of degradation. In some cases, a ladder of peaks among the lowest-molecular-weight DNA was seen (Figure 2C), which is evidence of necrotic or apoptosis-like damage due to enzymatic cleavage of chromatin between nucleosomes.

## 4. Discussion

DNA in fungal sporing bodies collected from nature may be damaged prior to collection by insects, collembolans, worms, and parasitic fungi, during collection and transport due to inadequate control of innate DNAase and acidity, during isolation by shearing, and at any stage by exposure to free radicals. Damage prior to collection leading to necrosis was minimised by selecting young, apparently intact specimens. Damage during collection and transport was minimised by rapid dehydration of tissue by slicing into isopropanol and by including sodium carbonate-impregnated filter paper slips to neutralise free acid. Shearing into shorter lengths during isolation was minimised by removing soluble contaminants while DNA was protected within chromatin, and by minimising exposure to shearing forces once the DNA was in solution. We chose not to add mercaptoethanol to buffers to moderate damage by free radicals during the extraction as, perversely, at low concentrations mercaptoethanol can promote free-radical damage. Instead, we rely on cell debris as a free-radical sink, providing protection for the DNA

Damage to fungal sporing bodies from insects, springtails, parasitic fungi, and the like is expected to cause local necrosis and DNA fragmentation. In the early stages of such decay, DNA will preferentially be digested between nucleosomes, leading to fragmentation into lengths incrementing with a periodicity of about 200 bp, giving a detectable banding pattern in gel electrophoresis [10]. A smear of all lengths on a gel may also reflect the action of acids weakening the DNA helix by depurination [11,12] or free-radical damage generating single-strand breaks [13], both of which lead readily to two-strand breaks. The addition of carbonate strips to the isopropanol at collection is a precaution against depurination. As flagged in the methods section, it is wise to use subdued laboratory light, particularly during the more extended steps of DNA purification. This is because free-radical generation from oxygen and other molecules occurs in the presence of light, particularly when coloured ions such as iron and pigments from tissue extracts are also involved [13].

Phenol is a reliable method for removing proteins and hydrophobic peptides, as well as cell debris in this application. However, phenol is toxic and corrosive, requiring safe handling protocols. All traces must be removed from the final DNA product as it interferes with yield estimations and inhibits enzymes used in PCR, sequencing, and other downstream processes. Phenol enters the matrix of plastic, and thus of tubes and pipetting devices, and then diffuses out to contaminate fluids. Hence, it is wise to reserve a specific set of pipettes for use with phenol or to decontaminate them under high vacuum.

In some cases, the extreme diversity of metabolites that may be encountered in fungi are difficult to remove from DNA. For example, *Entoloma rubromarginatum* produces a near-black, purple pigment, with properties akin to Indigo, Woad, or Tyrian purple, that co-purified with DNA. Isopropanol washes were ineffective, and while dialysis against SDS/Na_2_CO_3_/2-mercaptoethanol did extract pigment, the DNA was degraded.

Axenically cultured mycelium is the preferred starting material for preparing DNA for long-read sequencing. However, many fungi, particularly ectomycorrhizal macrofungi, remain impracticable to culture, and so material collected from nature, exposed to grazing and mechanical damage, must be used. Here, we offer a system for the collection of material suitable for use in remote locations where cryostorage is impractical, and a processing method that often succeeds in yielding DNA suitable for long-read sequencing. Failures are usually attributable to damaged starting material and sometimes to the presence of unusual metabolites. It is important to take care in selecting tissue without visible damage during collection. However, should a preparation fail to satisfy quality requirements and recollection not be feasible, resampling of collected material may avoid damaged tissue containing fragmented DNA and result in useable DNA preparations.

## 5. Reagents

**Sodium carbonate strips**: 1.5 cm × 5 cm strip of Whatman 3 MM filter paper that had been impregnated with 10% sodium carbonate and dried.

**HAE** **buffer.** 10× stock (50 mL)
  0.6 M K^+^ as KCl 2.236 g
  0.15 M Na^+^ as NaOH 0.30 g
  0.15 M HEPES as free acid 1.798 g
  20 mM EDTA free acid 292 mg
  1.5 mM Spermine tetra HCl 26.1 mg
  5.0 mM Spermidine HCl 63.7 mg
  Adjust to pH 7.5 with HCl or NaOH

**RNAase** 3 mg/mL in water. Activate by heating to 99 °C for 5 min and cool on ice.

**Trypsin** 10 mg/mL in water was chosen over protease K to reduce the release of carbohydrate polymers from glycoproteins. Either make fresh or store as aliquots at −80 °C. A simple check for the absence of inhibitors can be made by placing a drop of the fungal suspension on an exposed and developed film emulsion. Incubate in a humid chamber for 30 min and rinse with water. Active protease digests a hole in the emulsion.

**TTE buffer** (100 mM tetra-Tris EDTA) EDTA acid 2.92 g Tris 4.85 g in 100 mL. The pH does not require adjustment.

5%. **SDS**, sodium dodecyl sulphate (sodium lauryl sulphate) in 100 mM TTE buffer.

**3.0 M sodium acetate** made pH 5.2 with acetic acid.

**Phenol**. Two-phase phenol can be purchased under various alkaline buffers. Alternately, mix 8.74 g near-white phenol crystals with 2.2 mL 100 mM TTE and 1.1 mL water. Use the lower phenol phase only.

## Figures and Tables

**Table 1:** Number of collections and DNA preparations made of macrofungi in attempts to obtain genomes from Australian and New Zealand macrofungi. The absence of a JGI genome acronym indicates failure of all DNA preparations to meet either local or JGI quality control. Asterisks indicate that size selection electrophoresis was required to remove low-molecular-weight DNA. † Pileate-stipitate morphology refers to mushroom-like fungi that release spores forcibly for aerial dispersal. Sequestrate fungi include secotioid, gasteroid and truffle-like fungi with enclosed sporing bodies. Generic names in square brackets are former segregate genera for sequestrate species. Fungi were collected under permits: Scientific research permits U25406-13 and E26287-3, Department of Environment, Water and Natural Resources, South Australian Government. Authority to take Plants for Scientific Purposes FL 16065, Tasmania, Department of Primary Industries, Parks, Water and Environment. And DOC 65468-FLO, Ngāi Tahu Research Consultation Committee references 5709_20405, _20880 and _24045.

Family	Species	JGI Genome Acronym	BUSCO odb10%	CEGMA Capture %	Collection Number	Voucher	Morphology †	Collections Sampled	Times Resampled
*Amanitaceae*	*Amanita* [*Torrendia*] aff. *grandis*	Amagr1		98.9	KIS10	TL2892	sequestrate	2	
	*Amanita* aff. *conicoverrucosa*	Amapyr1		99.3	KIS12	PSC4683	pileate-stipitate	1	
	*Amanita* [*Torrendia*] *arenaria*				KIS16	PSC4900	sequestrate	1	
*Bolbitiaceae*	*Descolea maculata*	Descmac1 *		98.7	SA3	PSC4691	pileate-stipitate	1	1
*Cortinariaceae*	*Cortinarius abnormis*	Corab1 *		97.8	TAS10	PSC4364	pileate-stipitate	2	2
	*Cortinarius* [*Thaxterogaster*] aff. *campbelliae*	Corcam1		98.7	TAS5 culture	PSC4363	sequestrate	1	
	*Cortinarius* [*Thaxterogaster*] aff. *campbelliae 2*				KIS2	TL2885	sequestrate	2	
	*Cortinarius archeri*	Corarc1		98.5	SA1	PSC4685 TL3131	pileate-stipitate	1	
	*Cortinarius austrovenetus*	Coraus1		98.3	BRI184	BRI184	pileate-stipitate	2	
	*Cortinarius clelandii*				KIS9	PSC4599	pileate-stipitate	2	
	*Cortinarius globuliformis*				SA7	PSC4793a	sequestrate	1	
	*Cortinarius persplendidus*				KIS13	TL3124	pileate-stipitate	1	1
	*Cortinarius* sp. KIS14				KIS14	PSC4866	sequestrate	1	
	*Cortinarius* sp. KIS3	CorKIS3_1		98.9	KIS3	TL2766	pileate-stipitate	1	
	*Cortinarius* [*Protoglossum*] sp. TAS3	CorTAS3_1 *	96.2	98.9	TAS3	PSC4653	sequestrate	1	
	*Cortinarius* [*Thaxterogaster*] sp. KIS5				KIS5	TL2842	sequestrate	2	
*Entolomataceae*	*Entoloma* [*Richoniella*] *gasteromycetoides*	Entgas1			TAS9	PSC4662	sequestrate	1	
	*Entoloma rubromarginatum*				KIS17	PSC4923	pileate-stipitate	1	1
*Hydnangiaceae*	*Laccaria* [*Hydnangium*] aff. *carneum*	Hydca1 *	96.7		KIS1	TL2784	sequestrate	5	
	*Laccaria lateritia*				SA4	PSC4786	pileate-stipitate	1	1
	*Laccaria* sp. LAT2	LaccKIS6_1	98.9		KIS6	TL2844	pileate-stipitate	2	1
*Russulaceae*	*Lactarius* [*Zelleromyces*] sp. SA13				SA13	PSC4907	sequestrate	3	
	*Lactarius eucalypti*				SA9	PSC4799	pileate-stipitate	2	1
	*Russula* [*Cystangium*] aff. *sessile*				SA5	TL3400	sequestrate	1	
	*Russula* [*Cystangium*] *seminuda*	Russem1 *		98.5	TAS1	PSC4341	sequestrate	1	
	*Russula* sp. TAS8	Rusnee1 *		98.9	TAS8	PSC4658	pileate-stipitate	1	1
	*Russula* sp. SA2	RusSA2_1		98.5	SA2	PSC4686 TL3132	pileate-stipitate	1	
	*Russula* sp. KIS8R	RusKIS8R_1 *	95.9		KIS8R	nil	sequestrate	2	
	*Russula* sp. KIS4	RusKIS4_1_1		98.5	KIS4	TL3112	pileate-stipitate	1	1
*Serpulaceae*	*Austropaxillus* sp. SA11				SA11	TL3508	pileate-stipitate	1	
	*Austropaxillus muelleri*				KIS11	PSC4520	pileate-stipitate	1	
*Strophariaceae*	*Clavogaster virescens*	Clavir1		99.1	Mycota036	OTA70526	sequestrate	1	
	*Clavogaster* sp. ‘whakapapa’	Clawha1		99.1	Mycota032	OTA70370	sequestrate	1	
	*Hypholoma australianum*				TAS11	PSC4663	pileate-stipitate	1	1
	*Leratiomyces ceres*	Lerce2	95.8		Mycota028	OTA70338	pileate-stipitate	1	
	*Leratiomyces erythrocephalus*	Lerer2	95.6		Mycota031	OTA70433	sequestrate	1	
	*Psilocybe subaeruginosa*	Psisu1		99.3	BRI183	BRI183	pileate-stipitate	1	
	*Stropharia rugosoannulata*	Strrug1		99.6	Mycota018	nil	pileate-stipitate	1	

## Data Availability

Genomes arising in this project are available at https://mycocosm.jgi.doe.gov/mycocosm/home (accessed on 2 June 2025).
